# Effect of training using high-versus low-fidelity simulator mannequins on neonatal intubation skills of pediatric residents: a randomized controlled trial

**DOI:** 10.1186/s12909-022-03572-8

**Published:** 2022-06-25

**Authors:** Heidi Al-Wassia, Maha Bamehriz, Gamal Atta, Hamada Saltah, Abeer Arab, Abdulaziz Boker

**Affiliations:** 1 Department of Pediatrics, Division of Neonatology, Faculty of Medicine, King Abdulaziz University, Jeddah, Saudi Arabia; 2 King Abdulaziz University Hospital, Jeddah, Saudi Arabia; 3 Department of Anesthesia and Critical Care, Faculty of Medicine, King Abdulaziz University, Jeddah, Saudi Arabia; 4 Clinical Skills and Simulation Center, King Abdulaziz University, Jeddah, Saudi Arabia; 5 Anesthesiology Services Section, King Abdulaziz University, Jeddah, Saudi Arabia

**Keywords:** Simulation, Fidelity, Intubation, Neonate

## Abstract

**Background:**

Mounting evidence supports the effective acquisition of skills through simulation-based training including intubation skills of neonates. Our aim is to compare the effect of using high- versus low-fidelity mannequin simulation-based training on the acquisition and retention of neonatal intubation skills by junior pediatric residents.

**Methods:**

Randomized controlled trial involving first- and second-year pediatric residents from two centers in Jeddah, Saudi Arabia.

**Results:**

Twenty-eight junior pediatric residents (12 low- and 16 high-fidelity mannequins) completed the study. A significantly greater number of residents achieved and retained the required skills after completing the training course in both arms. There was no significant difference in the achieved skills between residents trained on high- versus low-fidelity mannequins at the baseline, immediately after training, and at 6–9 months after training.

**Conclusion:**

Simulation-based training resulted in improving pediatric residents’ intubation skills regardless of the level of fidelity.

## Background

Approximately 10% of newborns require some assistance to begin breathing at birth, and approximately 1% need extensive resuscitative measures to survive [1]. Optimal care provided during the first few minutes of life plays a significant role in reducing neonatal morbidity and mortality [2, 3]. Therefore, the Accreditation Council for Graduate Medical Education (ACGME) currently mandates the completion of formal training via the neonatal resuscitation program (NRP) by all pediatric residents [4]. Neonatal resuscitation is defined as the set of interventions at the time of birth to support the establishment of breathing and circulation [5].

Neonatal endotracheal intubation is an essential skill that every pediatrician must be trained to perform. Neonates are often exposed to more than one intubation attempt before the endotracheal tube is successfully placed and that is not without adverse effects [6]. Moreover, residents’ opportunities to intubate a neonate have been shown to decline and therefore simulation-based training was suggested [7–9].

Fidelity in simulation-based training is classified into low-, medium-, and high-fidelity simulators [10–12]. The term ‘fidelity’ is used to refer to the degree of realism of a simulation. Low-fidelity simulator (LFS) mannequins allow practitioners to demonstrate basic knowledge and technical and behavioral skills with verbal prompts from instructors. A LFS mannequin may or may not include the ability for intubation, chest rise, or electrocardiographic tracings. Medium-fidelity simulators (MFSs) produce physiological responses on a computer screen but lack various cues essential for participants to engage in the simulated scenario. For example, a medium-fidelity mannequin can produce breath sounds but no corresponding chest rise. High-fidelity simulators (HFSs) provide the trainee with the cues necessary to interact with the patient as they would in a real clinical environment. High-fidelity simulators can demonstrate physiologic signals (e.g., heart sounds, breath sounds, pulses, oxygen saturation and blood pressure) for the trainee to act upon. In response to the changing physiologic feedback received from the mannequin, the trainee is challenged to apply the correct interventions such as the administration of oxygen, endotracheal intubation or chest drain insertion to improve the condition of the simulated mannequin.

High-fidelity simulation is a rapidly developing technology that has been in use for years in a variety of medical fields. A growing body of evidence supports the effective acquisition and retention of technical skills through HFS [13–15]. However, there are few studies describing the use of HFS in teaching neonatal resuscitation.

Our research question was the following: Among junior (first year) pediatric residents, would HFS training result in better neonatal intubation technical skills compared to LFS training? Answering this question can inform the supporting and financing bodies to whether selecting a training method that is more costly (HFS) is justified by a better outcome of improving pediatric trainees’ neonatal intubation skills and the retention of these skills towards enhanced patient care.

## Methods

This is a randomized controlled trial that was approved by the King Abdulaziz University Hospital (KAUH) Hospital’s Ethical Review Board (Reference No 471-lE) and registered in the ISCRTN registry (ISRCTN16251230) 21/03/2018. The study was carried out in accordance with the CONSORT 2010 statement guidelines for reporting parallel group randomized trials [16]. Our primary objective was to determine the effectiveness of using HFSs compared to LFSs in improving the technical skills to successfully intubate a neonate, defined as the passage of the endotracheal tube (ET) beyond the vocal cords. The secondary objectives included determining whether using HFSs will result in a shorter time taken to intubate a neonate and better retention of technical neonatal intubation skills compared to LFSs.

### Population

The study participants were all pediatric residents in their first year of postgraduate training in pediatrics recruited from two hospitals in Jeddah, Kingdom of Saudi Arabia (KSA), and agreed to participate in the study. A total of 28 pediatric residents from KAUH and Maternity and Children Hospital (MCH) were included in the study after signing an informed consent form. The pediatric residency program in Saudi Arabia is a 4-year National Training Program during which pediatric residents rotate through the Neonatal Intensive Care Unit (NICU) for 2 months in the first year and one month in the second, third and fourth years of their program. NRP training is not a requirement for pediatric residency programs in the two participating hospitals. Junior residents acquire skills to intubate a neonate through rotating in NICU in which a neonatal specialist or fellow will be present for all neonatal intubation attempts. There was no didactic intubation training or structured intubation apprenticeship in NICU in either hospital.

### Intervention

After recruitment, residents were randomly assigned to training on either a high-fidelity or low-fidelity mannequin. The assignment was achieved using a computer-generated random number that was placed in sequentially numbered concealed opaque envelopes by one of the simulation center organizers. The participating residents were first assessed at the baseline on their allocated mannequin, where they were asked to manage a standardized simulated scenario of a neonate requiring intubation. A training course was designed to improve the intubation skills of pediatric residents using the assigned mannequins and delivered by two NRP providers with more than ten-year experience in neonatology. The high-fidelity mannequin (SimBaby, Laerdal Medical Corporation, USA) is an advanced infant patient simulator with realistic airway anatomy and clinical functionality. It features airway opening by appropriate maneuvers, suctioning motion, anatomical landmarks of oropharyngeal and nasopharyngeal airways, and chest rise and oxygen saturation changes in response to intervention. The mannequin utilizes software-based medical model that was programmed to display changes in physical findings in response to progressing medical circumstances. The resident will have to continually evaluate the mannequin’s condition rather than relying on cues provided by an instructor [17, 18]. The low-fidelity mannequin is a standard plastic mannequin (ALS Baby, Laerdal Medical Corporation, USA) that allows passing ET tube and demonstrates chest rises in response to successful intubation. It does not, however, feature anatomical landmarks of the neonatal airway or give physiological cues such as oxygen saturation, heart rate and changes in CO_2_ detector color [17]. A one-hour introductory lecture outlining the indications for intubations, anatomical landmarks and steps taken to prepare for, perform and confirm successful intubation was given to both groups by one of the physicians assigned for training. The residents then had the chance to practice the steps of successful intubation on the assigned mannequin 2–3 times in the presence of the other residents in the group. Constructive feedback was provided to trainees in both groups after a successful intubation attempt. Each participant was re-evaluated using a standardized simulated scenario of intubating a neonate after training using a high-fidelity mannequin.

### Outcome

Two trained assessors who were certified NRP instructors independently evaluated the technical neonatal intubation skills of pediatric residents using a checklist of appropriate and inappropriate actions derived from the published literature at the baseline and both at the end of the training course and 6–9 months after completion of the course. The evaluators were blinded to the training intervention and different from the physicians who trained the residents. The technical skills were evaluated using a 13-item binary intubation checklist that was used in a previous study evaluating the skills needed to intubate a neonate, including preparing for the intubation, technical skills for positioning and inserting the endotracheal tube and confirming successful intubation [19]. The checklist scores in the study had an interrater reliability of 0.88 and correlated positively with the global rating scores (Spearman’s coefficient 0.68). The authors of the study gave permission to use their checklist. The time taken to intubate and ensure correct placement was documented (using a stopwatch) from the start of the intubation attempt until the trainee presented evidence of correct placement. A 5-point Likert global rating score (1 being the lowest and 5 being the highest score) was utilized by the evaluators to provide an overall assessment of resident’s capability to perform a neonatal intubation.

### Data analysis

IPM SPSS 22.0 (SPSS Inc, Chicago, IL) was used for the data analyses. Normally distributed data are presented as the mean ± SD and were compared between two groups using Student’s t-test. Repeated measures analysis of variance (ANOVA) was applied when the three periods of assessment were compared. Categorical parameters are presented as frequencies and percentages and were compared by applying the chi-squared test. The level of significance was specified at *p* < 0.05.

## Results

**Fig. 1:**
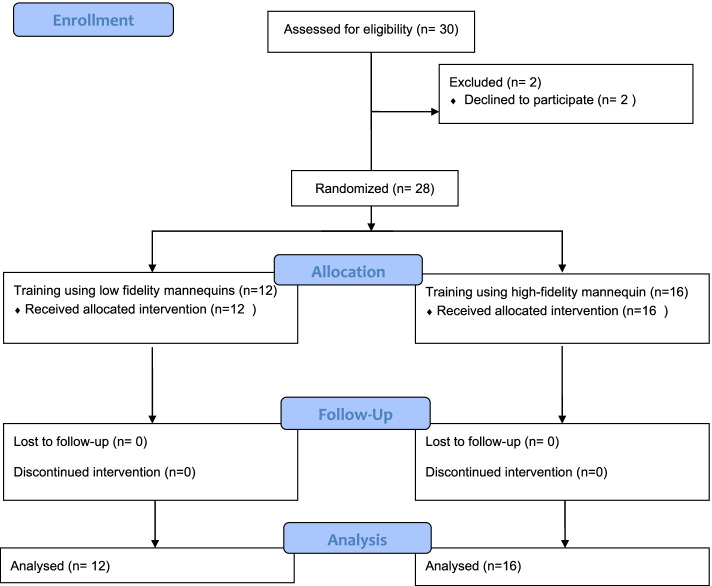
CONSORT Diagram for the Randomized Controlled Trial (RCT)

**Fig. 2:**
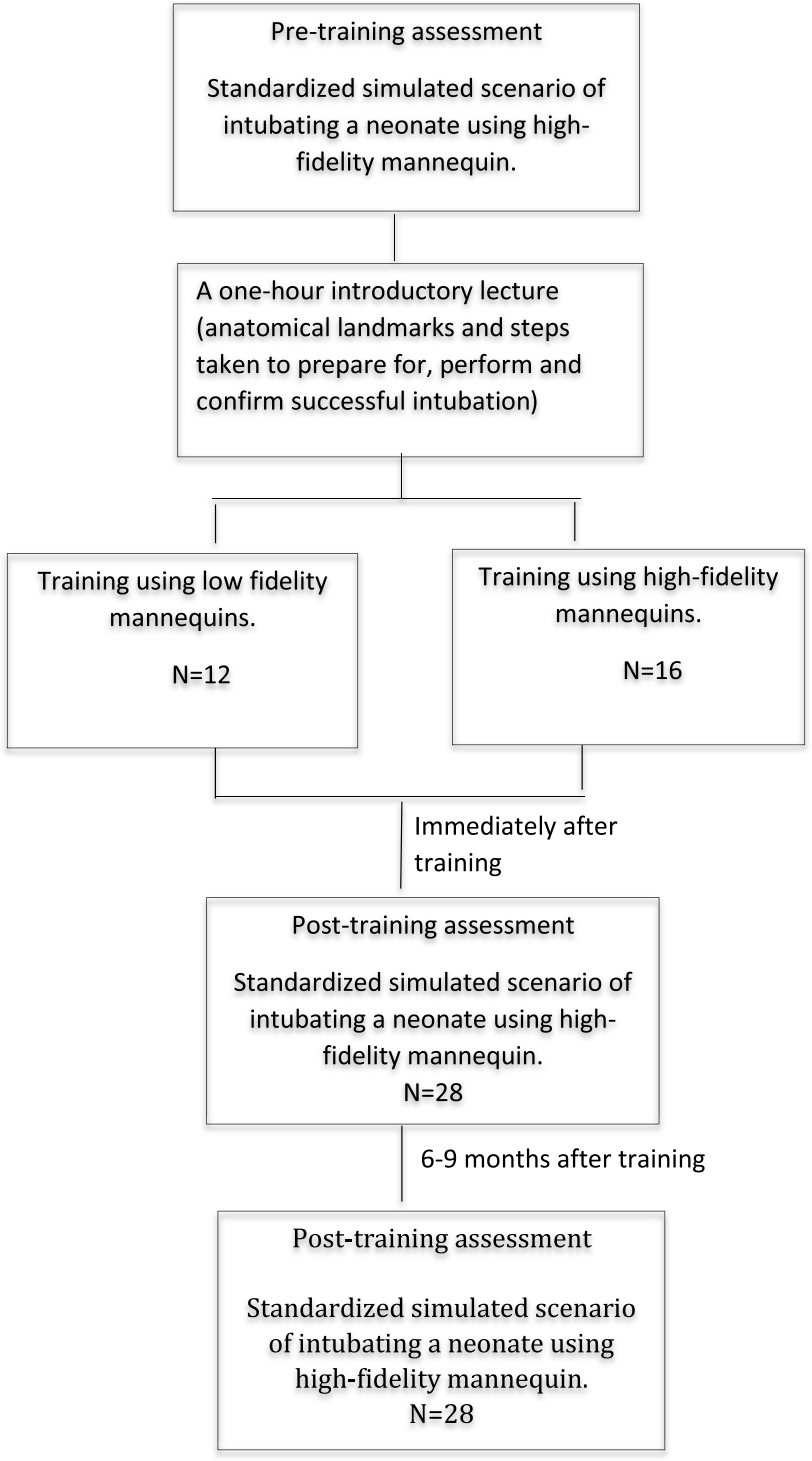
Flow diagram of participated residents

Thirty residents were invited to participate in the study (Fig. 1). Twenty-eight residents participated and completed the study. Twelve residents were allocated to the low-fidelity arm and sixteen to the high-fidelity arm (Fig. 2). There was no difference between participants in either arm regarding age, sex, training center, NRP certification and whether they intubated a neonate before the training course (Table 1). A significantly greater number of residents achieved the required skills immediately after the training course in both arms (Table 2). In addition, the achieved skills were retained when the residents were tested at 6–9 months after training (Table 2). There was no significant difference between the achieved skills between residents trained on high-fidelity mannequins compared to those trained on low-fidelity mannequins at the baseline, immediately after training, and at 6–9 months after training (Table 3).

**Table 1:** Demographic characteristics of groups at baseline

	Low fidelity Mannequin (*N* = 12)	High fidelity Mannequin (*N* = 16)
Age (years)	25.6 (0.67)	26 (1.9)
Male	4 (33.3)	2 (12.5)
Female	8 (66.7)	14 (87.5)
NRP	2 (16.7)	5 (31.3)
Intubation before training	1 (8.3)	2 (12.5)
Centre 1	5 (41.7)	8 (50.0)
Centre 2	7 (58.3)	8 (50.0)

Data are presented as n (%) or mean (SD)

**Table 2:** Comparison between intubation skills at baseline, immediately and 6–9 months after training for each training method

	Low fidelity (*N* = 12)				High fidelity (*N* = 16)			
	Baseline	Immediately after training	6–9 months after training	*P*-value	Baseline	Immediately after training	6–9 months after training	*P*-value
Select appropriate size tube	1 (8.3)	11 (91.7)	12 (100)	0.000	3 (18.8)	16 (100)	12 (75.0)	0.000
Ensures availability of all needed equipment	0	12 (100)	9 (75.0)	0.000	0	16 (100)	9 (56.3)	0.000
Pre-oxygenate	1 (8.3)	8 (6.7)	5(41.7)	0.013	1 (6.3)	7 (43.8)	10 (62.5)	0.004
Position the head	1 (8.3)	6 (50)	6 (50.0)	0.049	0	10 (62.5)	10 (62.5)	0.000
Insertion of blade correctly using left hand	5 (41.1)	7 (58.3)	9 (75.0)	0.254	5 (31.3)	14 (87.5)	12 (75.0)	0.002
Lifts handle forward	3 (25)	4 (33.3)	8 (66.7)	0.091	1 (6.3)	8 (50.0)	5 (31.3)	0.024
Visualization of vocal cords	2 (16.7)	7 (58.3)	10 (35.7)	0.004	2 (12.5)	8 (50.0)	7(43.8)	0.059
Suction appropriately	12 (100)	8 (66.7)	5 (41.7)	0.003	16(100)	4 (25.0)	7 (43.8)	0.013
Pass tube smoothly	1 (8.3)	8 (66.7)	4 (33.3)	0.012	1 (6.3)	8 (50.0)	5 (31.3)	0.024
Check position of tube	0	7 (58.3)	3 (25.0)	0.006	3 (18.3)	7 (43.8)	7 (43.8)	0.233
Check placement by auscultation	4 (33.3)	9 (75.0)	5 (41.7)	0.097	5 (31.3)	13 (81.3)	10 (62.5)	0.015
Successful intubation	2 (16.7)	6 (50)	4 (33.3)	0.223	2 (12.5)	7 (43.8)	9 (56.3)	0.083
**Overall Score**	1.25 (0.45)	3.08 (1.24)	3.33 (1.55)	0.000	1.19 (0.54)	2.75 (0.86)	3.25 (1.0)	0.000
**Duration of intubation (seconds)**	51.1 (22.9)	51.8 (48.55)	36.2 (13.14)	0.344	46.38 (19.9)	34.6 (19.27)	42.8 (14.8)	0.126

Data are presented as n (%) or mean (SD)

**Table 3:** Comparison between the two training methods in the achieved skills for intubating a neonate at baseline, immediately after training and 6–9 months after

	Baseline			Immediately after training			6–9 months after training		
	Low fidelity (*N* = 12)	High fidelity (*N* = 16)	*P*-value	Low fidelity (*N* = 12)	High fidelity (*N* = 16)	*P*-value	Low fidelity (*N* = 12)	High fidelity (*N* = 16)	*P*-value
Select appropriate size tube	1 (8.3)	3 (18.8)	0.436	11 (91.7)	16 (100)	0.240	12 (100)	12 (75)	0.061
Ensures availability of all needed equipment	0	0		12 (100)	16(100)	0.595	9 (75.0)	9 (56)	0.306
Pre-oxygenate	1 (8.3)	1 (6.3)	0.832	8 (6.7)	7 (43.8)	0.229	5 (41.7)	10 (62.5)	0.274
Position the head	1 (8.3)	0	0.240	6 (50.0)	10 (62.5)	0.508	6 (50.0)	10 (62.5)	0.508
Insertion of blade correctly using left hand	5 (41)	5 (31.3)	0.569	7 (58.3)	14 (87.5)	0.078	9 (75.0)	12 (75)	1.000
Lifts handle forward	3 (25)	1 (6.3)	0.161	4 (33.3)	8 (50.0)	0.378	8 (66.7)	5 (31.3)	0.063
Visualization of vocal cords	2 (16.7)	2 (12.5)	0.755	7 (58.3)	8 (50.0)	0.662	10 (35.7)	7 (43.8)	0.034
Suction appropriately	12 (100)	16 (100)	NA	8 (66.7)	4 (25.0)	0.027	5 (41.7)	7 (43.8)	0.912
Pass tube smoothly	1 (8.3)	1 (6.3)	0.832	8 (66.7)	8 (50.0)	0.378	4 (33.3)	5 (31.3)	0.907
Check position of tube	0	3 (18.3)	0.112	7 (58.3)	7 (43.8)	0.445	3 (25.0)	7 (43.8)	0.306
Check placement by auscultation	4 (33.3)	5 (31.3)	0.907	9 (75.0)	13(81.3)	0.690	5 (41.7)	10(62.5)	0.274
Successful intubation	2 (16.7)	2 (12.5)	0.755	6 (50.0)	7 (43.8)	0.743	4 (33.3)	9 (56.3)	0.393
**Overall score**	1.25 (0.5)	1.2 (0.5)	0.749	3.1 (1.2)	2.8(0.9)	0.407	3.3 (1.6)	3.3(1.0)	0.840
**Duration of intubation (seconds)**	51.1(22.9)	46.4(19.9)	0.567	51.8 (48.6)	34.6(19.3)	0.205	36.2(13.1)	42.8(14.8)	0.229

Data are presented as n (%) or mean (SD)

## Discussion

We found no difference in the effect of high- versus low-fidelity mannequins on the acquisition and retention of technical skills necessary to intubate a neonate by junior pediatric residents from the two academic centers. However, we concluded that training junior pediatric residents using either simulator mannequins resulted in improved intubation skills both immediately and 6–9 months after training.

Experiential learning methods, as the case in simulation-based training, were shown to result in deeper learning and better retention [13, 14]. Experiential learning theory proposes that knowledge is generated through the transformation of experience [20]. In his work on experiential learning, Kolbe described how the learner moves through four stages of adaptive learning modes: more concrete experience, reflective observation, abstract conceptualization, and active experimentation. Dewey was the first to introduce the concept of experience plus reflection equals learning that was considered to be the foundation of simulation-based learning [15]. The quality and amount of active experimentation is judged by the degree to which it reproduces reality. Experiential learning helps individuals understand their strengths and weaknesses; gives the opportunity to apply skills for assessment, examination and evaluation and encourages critical thinking and the development of proficiency in an environment where errors do not have dire consequences. In theory, through experiential learning, trainees retain not only knowledge but also its practical application and how to use it when needed. As a result, novice trainees will move towards becoming experts.

Evidence supporting the use of HFS in neonatal resuscitation and the associated acquisition of technical skills does not agree with the evidence from other medical fields. Similar to our results, Campbell et al. reported that although pediatric residents randomly assigned to the HFS gave better satisfaction scores than those assigned to the LFS, they did not have improved written scores or intubation times [13]. In a small randomized controlled trial of ten second- and third-year pediatric residents, HFS training did not result in a better intubation technique or actual procedural success [20]. More recently, Finan et al. compared the effects of HFS versus LFS in a randomized trial of sixteen neonatal fellows and found no difference in stress measures between the two modalities [21]. Regarding the retention of the learned skills in simulation-based training, studies have shown degradation of the technical skills acquired in certification courses by health professionals over time, which can have significant effects on patient outcomes [19, 22–25].

We would like to acknowledge some of the limitations of this study. The small sample size limits the significance and generalizability of our study as it was a convenience sample of all junior pediatric residents from the two centers which agreed to participate. The high-fidelity mannequin was used both to train and to assess; therefore, additional training exposure might have introduced additional benefits, but that was not statistically evident. Moreover, the residents were allowed to practice 2–3 times on the simulator which might not be enough for robust skill acquisition. We did not record the number of practice repetitions residents did before their evaluation session which may have impacted the results of this study. Furthermore, residents’ experience during the lag time between the training course and assessment of the retention of learned intubation skills might differ as they do not all rotate through the NICU at a fixed time, which might expose some of them to more intubation trials than others. We recorded the intubation trials before the training course but not before the assessment of the retention of skills. We minimized the bias by structuring the training course in a way that only the type of mannequin was the changing variable and blinded the assessors to the intervention applied to strengthen the internal validity of the study. Additionally, the effect on improving noncognitive skills such as attentiveness, perseverance, and teamwork was not part of the current study. Although simulation teaching has been demonstrated to lead to improved educational outcomes, transfer of these skills from simulation to the real clinical world, and to the benefits of clinical patient outcomes will need to be studied.

In conclusion, simulation-based training resulted in improving pediatric residents’ intubation skills regardless of the level of fidelity. The benefit of training on a high-fidelity mannequin will need to be further assessed in real-life intubations to determine its effect on the management and clinical outcome of neonatal intubation.

## Data Availability

The datasets used and/or analyzed during the current study are available from the corresponding author on reasonable request.

## Abbreviations

ACGME: Accreditation Council for Graduate Medical Education
ANOVA: Analysis of variance
ET: Endotracheal tube
HFS: High-fidelity simulator
KAUH: King Abdulaziz University Hospital
KSA: Kingdom of Saudi Arabia
LFS: Low-fidelity simulator
NCH: Maternity and Children Hospital
MFS: Medium fidelity simulator
NICU: Neonatal Intensive Care Unit
NRP: Neonatal Resuscitation Program

## References

[1] J WGZ. Textbook of Neonatal Resucitation (NRP). 7th Ed. ed2016.

[2] Duran R, Aladag N, Vatansever U, Sut N, Acunas B (2008). The impact of Neonatal Resuscitation Program courses on mortality and morbidity of newborn infants with perinatal asphyxia. Brain Dev.

[3] Patel D, Piotrowski ZH, Nelson MR, Sabich R (2001). Effect of a statewide neonatal resuscitation training program on Apgar scores among high-risk neonates in Illinois. Pediatrics.

[4] Education ACfGM. 2021 [Available from: https://www.acgme.org/specialties/pediatrics/overview/.

[5] Black RE, Cousens S, Johnson HL, Lawn JE, Rudan I, Bassani DG (2010). Global, regional, and national causes of child mortality in 2008: a systematic analysis. Lancet.

[6] Foglia EE, Ades A, Sawyer T, et al. Neonatal Intubation Practice and Outcomes: An International Registry Study. Pediatrics. 2019;143(1):e20180902. 10.1542/peds.2018-0902 PMC6317557 30538147

[7] Gozzo YF, Cummings CL, Chapman RL, Bizzarro MJ, Mercurio MR (2011). Who is performing medical procedures in the neonatal intensive care unit?. J Perinatol.

[8] Downes KJ, Narendran V, Meinzen-Derr J, McClanahan S, Akinbi HT (2012). The lost art of intubation: assessing opportunities for residents to perform neonatal intubation. J Perinatol.

[9] Lopreiato JO, Sawyer T (2015). Simulation-based medical education in pediatrics. Acad Pediatr.

[10] Maran NJ, Glavin RJ (2003). Low- to high-fidelity simulation - a continuum of medical education?. Med Educ.

[11] Epps C, White M, Tofil N (2013). Mannequin Based Simulators. In A. I. Levine, S., DeMaria, A. Schwartz, & A. Sim (Eds).

[12] Palaganas J, Ulrich B, Mancini B (2020). Mastering simulation: A handbook for success. : Sigma.

[13] Campbell DM, Barozzino T, Farrugia M, Sgro M (2009). High-fidelity simulation in neonatal resuscitation. Paediatr Child Health.

[14] Thomas EJ, Williams AL, Reichman EF, Lasky RE, Crandell S, Taggart WR (2010). Team training in the neonatal resuscitation program for interns: teamwork and quality of resuscitations. Pediatrics.

[15] Yaeger KA, Halamek LP, Coyle M, Murphy A, Anderson J, Boyle K (2004). High-fidelity simulation-based training in neonatal nursing. Adv Neonatal Care.

[16] Schulz KF, Altman DG, Moher D, Group C (2010). CONSORT 2010 statement: updated guidelines for reporting parallel group randomized trials. Ann Intern Med.

[17] Lopreiato JOE, Downing, D., Gammon, W., Lioce, L., Sittner, B., Slot, V., Spain, A. E. (Associate Eds.), and the Terminology & Concepts Working Group. Healthcare simulation dictionary 2016 [Available from: Retrieved from http://www.ssih.org/dictionary. .

[18] Deutsch ES (2008). High-fidelity patient simulation mannequins to facilitate aerodigestive endoscopy training. Arch Otolaryngol Head Neck Surg.

[19] Bismilla Z, Finan E, McNamara PJ, LeBlanc V, Jefferies A, Whyte H (2010). Failure of pediatric and neonatal trainees to meet Canadian Neonatal Resuscitation Program standards for neonatal intubation. J Perinatol.

[20] Sharara-Chami R, Taher S, Kaddoum R, Tamim H, Charafeddine L (2014). Simulation training in endotracheal intubation in a pediatric residency. Middle East J Anaesthesiol.

[21] Finan E, Bismilla Z, Whyte HE, Leblanc V, McNamara PJ (2012). High-fidelity simulator technology may not be superior to traditional low-fidelity equipment for neonatal resuscitation training. J Perinatol.

[22] Smith KK, Gilcreast D, Pierce K (2008). Evaluation of staff's retention of ACLS and BLS skills. Resuscitation.

[23] Kaczorowski J, Levitt C, Hammond M, Outerbridge E, Grad R, Rothman A (1998). Retention of neonatal resuscitation skills and knowledge: a randomized controlled trial. Fam Med.

[24] Grant EC, Marczinski CA, Menon K (2007). Using pediatric advanced life support in pediatric residency training: does the curriculum need resuscitation?. Pediatr Crit Care Med.

[25] Hunt EA, Fiedor-Hamilton M, Eppich WJ (2008). Resuscitation education: narrowing the gap between evidence-based resuscitation guidelines and performance using best educational practices. Pediatr Clin North Am..

